# Targeting metabolism with arsenic trioxide and dichloroacetate in breast cancer cells

**DOI:** 10.1186/1476-4598-10-142

**Published:** 2011-11-18

**Authors:** Ramon C Sun, Philip G Board, Anneke C Blackburn

**Affiliations:** 1 Molecular Genetics Group, Department of Translational Biosciences, John Curtin School of Medical Research, Building 131, Australian National University, P.O. Box 334, Canberra ACT 0200, AUSTRALIA; 2 Department of Radiation Oncology, Stanford School of Medicine, Stanford CA 94305 USA

**Keywords:** Dichloroacetate, breast cancer, electron transport chain, mitochondria, arsenic trioxide

## Abstract

**Background:**

Cancer cells have a different metabolic profile compared to normal cells. The Warburg effect (increased aerobic glycolysis) and glutaminolysis (increased mitochondrial activity from glutamine catabolism) are well known hallmarks of cancer and are accompanied by increased lactate production, hyperpolarized mitochondrial membrane and increased production of reactive oxygen species.

**Methods:**

In this study we target the Warburg effect with dichloroacetate (DCA) and the increased mitochondrial activity of glutaminolysis with arsenic trioxide (ATO) in breast cancer cells, measuring cell proliferation, cell death and mitochondrial characteristics.

**Results:**

The combination of DCA and ATO was more effective at inhibiting cell proliferation and inducing cell death than either drug alone. We examined the effect of these treatments on mitochondrial membrane potential, reactive oxygen species production and ATP levels and have identified new molecular mechanisms within the mitochondria for both ATO and DCA: ATO reduces mitochondrial function through the inhibition of cytochrome C oxidase (complex IV of the electron transport chain) while DCA up-regulates ATP synthase β subunit expression. The potentiation of ATO cytotoxicity by DCA is correlated with strong suppression of the expression of c-Myc and HIF-1α, and decreased expression of the survival protein Bcl-2.

**Conclusion:**

This study is the first to demonstrate that targeting two key metabolic hallmarks of cancer is an effective anti-cancer strategy with therapeutic potential.

## Introduction

Arsenic trioxide (ATO) has been used as a therapeutic agent for over 2000 years. Originating from China [1], it is currently being used against acute promyeloid leukemia (APL) in patients who have relapsed following all-trans-retinoic acid/anthracycline therapy and is being promoted for first line therapy of de novo APL [2-4]. ATO is known as a hyper-reactive molecule and could potentially bind to thiol groups in many proteins [2,5]. Its ability to bind to the thiol-rich, mutant protein PML-RAR-α produced from a chromosome translocation in APL has made it an effective drug in APL [2,5,6]. ATO has been shown to induce apoptosis in a variety of cancer cell lines *in vitro *and *in vivo *[7,8], but it has been difficult to consider ATO for clinical use in tumor types other than APL due to the lack of knowledge of the molecular targets that result in its cytotoxicity. In the past 10 years, physiological changes within cancer cells in response to ATO treatment have been well characterized, and many clinical trials for new applications of ATO are underway [5]. ATO has been proposed as a mitochondrial toxin [9]. ATO can depolarise the mitochondrial membrane potential (MMP) [10], increase intracellular reactive oxygen species (ROS) production [8], and induce apoptosis [8]. The proposed target for ATO that can achieve these phenotypic changes is the mitochondrial transition pore (MTP) [11]. ATO has been shown to induce the opening of the MTP, which induces cytochrome c release and is proposed to dissipate the MMP and increase ROS release from the mitochondria [12]. More recently, the thioredoxin system, in particular thioredoxin reductase, has also been identified as a target of ATO that may contribute to increased oxidative stress and altered redox signalling after ATO treatment of cancer cells [9,13].

The Warburg effect is a wide spread phenomenon that has been identified in over 90% of all tumor forms. Cells that exhibit the Warburg effect take on alternative routes of energy homeostasis to maintain their proliferative phenotype [14]. Nobel laureate Dr Otto Warburg stated that cancer cells rely on glycolysis or substrate phosphorylation to generate ATP, and suppress their mitochondrial activities [15]. With more advanced technologies, recent studies have confirmed the ATP production aspect of the Warburg hypothesis but revealed that mitochondrial activity is not suppressed in cancer cells. Instead mitochondria play vital roles in providing substrates to maintain cell division [16].

The anti-cancer effect of reversing the Warburg effect has been described recently and an old drug dichloroacetate (DCA), which can redirect ATP synthesis from glycolysis to oxidative phosphorylation, has demonstrated good anti-cancer activity both *in vitro *[17-19] and *in vivo *[20-23]. DCA is a pyruvate dehydrogenase kinase inhibitor, and results in increased pyruvate dehydrogenase activity [19]. This leads to the increased conversion of pyruvate to acetyl-CoA rather than lactic acid as described by the Warburg effect, and stimulates mitochondrial respiration by increasing the supply of acetyl-CoA. Consequently, after DCA treatment, cancer cells showed increased levels of ROS, depolarization of the MMP *in vitro *and increased apoptosis both *in vitro *and *in vivo *[17,20].

As DCA can redirect substrates into mitochondrial respiration and ATP production, it could have a synergistic activity with anti-cancer drugs that impair mitochondrial activity. We propose that by reversing the glycolytic phenotype with DCA and directing more pyruvate into mitochondrial oxidative phosphorylation, while simultaneously targeting the mitochondria with ATO, a severe disruption of energy homeostasis will occur within cancer cells. In this study we show that in combination DCA and ATO work together to inhibit the growth of breast cancer cell lines *in vitro*. Further, we have identified new molecular mechanisms within the mitochondria that can contribute to the cytotoxicity of ATO providing additional support for the use of ATO against solid tumours.

## Materials and methods

### Reagents

JC-1, CFSE and H_2_DCFDA were from Invitrogen (Carlsbad, CA, USA), CellTiter-Glo and Caspase-Glo assay kits were purchased from Promega Co (San Luis, CA, USA). The rest of the chemicals were purchased from Sigma Co (St. Louis, MO, USA).

### Cell culture

Cell lines were obtained from the following sources in the years indicated. Dr Anna DeFazio, Westmead Millenium Institute, Sydney, Australia: T-47D (2003), BT-20 (2003), MCF-10A (2005) and MCF-10AT1 (2005); Prof Chris Parish, Australian National University, Canberra, Australia: 13762 MAT (2007), MDA-MB-468 (2003) and MDA-MB-231 (2003). The cell lines have appearances consistent with published morphologies, but have not been authenticated recently. Human breast epithelial carcinoma cells (T-47D) were grown in RPMI 1640 medium supplemented with 10% fetal bovine serum in the presence of 0.1% PSN (3% penicillin, 5% streptomycin and 5% neomycin). BT-20, MDA-MB-231, MDA-MB-468, and 13762 MAT cell lines were maintained in DMEM/F-12 medium supplemented with 10% FBS and 2 mM L-glutamine. DMEM/F-12 medium with 25% horse serum, 0.01% EGF, 0.28 IU/ml insulin, 0.01% cholera toxin and 0.5 μg/ml hydrocortisone used for MCF-10A and MCF-10AT1 cells. All of the cell lines were maintained at 37°C in 5% CO_2_.

### Cell viability

For the assessment of cell viability, cells were plated in 96 well plates at a density of 3000 cells per well and 8 wells per group. Following exposure to DCA and ATO for 24 to 72 hours, cells were incubated for 3 hours with neutral red (30 μg/ml) in fresh media, then washed with PBS, followed by the addition of lysis buffer (acetic acid/methanol, 80%/20%) and the absorbance at 540 nm was recorded. Results are expressed as mean ± S.D, calculations were performed using the Prism software package, ANOVA with Tukey post test was applied and P < 0.05 was considered to be statistically significant. Experiments were performed at least three times and data presented are of one representative experiment.

### Cell proliferation

T-47D cells were harvested and resuspended in 1 ml of RPMI medium. Cells were labelled by the addition of 1 ml of PBS containing 5 μM The carboxyfluorescein succinimidyl ester (CFSE), followed by 5 min incubation at room temperature. Labelled cells were then washed twice, counted and seeded at 10^5 ^cells/well in a 12 well plate. On the day of analysis, T-47D cells were harvested and washed twice with PBS and then CFSE intensity was examined by FACS. Results are expressed as mean ± S.D (n = 3), calculations were performed using the Prism software package, ANOVA with Tukey post test was applied and P < 0.05 was considered to be statistically significant. Experiments were performed at least three times and data presented are of one representative experiment.

### Cell death

Apoptosis was quantitated by flow cytometry after staining cells with FITC-labelled Annexin-V (AV) (Invitrogen Co.) and propidium iodide (PI). After drug treatment, T-47D cells were harvested and centrifuged at 1200 rpm for 5 min; the pellets were washed twice with PBS and then resuspend in 100 μl of Annexin-V binding buffer (0.14 M NaCl, 2.5 mM CaCl_2_, 0.01 M HEPES pH 7.4). Annexin-V (1 μL) and 5 μl of PI (50 μg/ml) were added to the samples and incubated in the dark for 15 min. Samples were kept on ice after incubation until FACS analysis was performed. Results are expressed as mean ± S.D (n = 3), calculations were performed using the Prism software package, ANOVA with Tukey post test was applied and P < 0.05 was considered to be statistically significant. Experiments were performed at least three times and data presented are of one representative experiment.

### ROS generation

For the assessment of intracellular ROS levels, cells were plated in 12 well plates with cell density of 1 × 10^5 ^cells per well and treated with drugs for 12 hours. 2', 7'-dihydrochlorofluroresceinacetate (H_2_DCFDA) was added to the medium at a final concentration of 10 μM and the cells were allowed to stain for 1 hour in the dark. After H_2_DCFDA staining, cells were trypsinized, washed twice and resuspended in 100 μl of PBS. H_2_DCFDA intensity was examined using FACS. Results are expressed as mean ± S.D (n = 3), calculations were performed using the Prism software package, ANOVA with Tukey post test was applied and P < 0.05 was considered to be statistically significant. Experiments were performed at least three times and data presented are of one representative experiment.

### ATP concentration and Caspase activity

Internal ATP level and caspase activity in T-47D cells was assessed using CellTiter-Glo and Caspase-Glo 3/7 assay kits (Promega Corp., Madison, WI) according to the manufacturer's instructions. T-47D cells were cultured in the absence and presence of drugs for 12 hours in white opaque 96 well plates (4 wells per group). Equal volumes of CellTiter-Glo reagents were added after which the samples were incubated for 15 min on a shaker at room temperature. Luminescence was recorded using the Glomax micro-plate luminometer (Promega Co., Madison, WI) under the preset CellTiter protocol. Results are expressed as mean ± S.D (n = 4), calculations were performed using the Prism software package, ANOVA with Tukey post test was applied and P < 0.05 was considered to be statistically significant. Experiments were performed at least three times and data presented are of one representative experiment.

### Mitochondrial membrane potential

Similarly to the measurement of ROS, cells were plated in 12 well plates with cell density of 1 × 10^5 ^cells per well and treated with drugs for 12 hours. 5, 5', 6, 6'-tetrachloro-1, 1', 3, 3'-tetraethylbenzimidazol-carbocyanine iodide (JC-1) was added in the medium at a final concentration of 0.2 μM and the cells were allowed to stain for 30 min in the dark. After JC-1 staining, cells were trypsinized, washed twice with PBS and resuspended in 100 μl of PBS. JC-1 intensity was examined using FACS. Results are expressed as mean ± S.D (n = 3), calculations were performed using the Prism software package, ANOVA with Tukey post test was applied and P < 0.05 was considered to be statistically significant. Experiments were performed at least three times and data presented are of one representative experiment.

### Cytochrome C oxidase activity

Cytochrome C oxidase assay was assessed based on the method previously published [24]. Briefly, after drug treatment in 96 well plates (8 wells per group), T-47D cells were permeabilized with 50 μl of 0.01% saponin, followed by the addition of 100 μl substrate medium (4 mM 3,3-diaminobenzidine tetrahydrochloride (DAB), 100 μM reduced cytochrome C, 2 μg/ml catalase in 0.1 M Na phosphate, pH 7.0). Absorbance at 450 nm was measured immediately after the addition of substrate medium and monitored over 30 min. Results are expressed as mean ± S.D (n = 8), calculations were performed using the Prism software package, ANOVA with Tukey post test was applied and P < 0.05 was considered to be statistically significant. Experiments were performed at least three times and data presented are of one representative experiment.

### PDH activity

PDH activity was measured using the MitoSciences PDH enzyme activity microplate assay kit (#MSP18, MitoSciences, Oregon USA). For measuring the effect of drugs on PDH activity in cells, T-47D cells were treated with drugs in media for 3 hrs, after which they were washed and resuspended in PBS. Cell extracts were prepared and assayed for PDH activity at a concentration of 15 mg protein/ml, according to the kit instructions. For measurement of direct inhibition of PDH by ATO, PDH was isolated from untreated T-47D cells by applying cell extracts to the antibody capture plate. After washing the plate, assay solution containing drug was added to the wells and then immediately assayed for activity PDH. Results are expressed as mean ± S.D (n = 4), calculations were performed using the Prism software package, ANOVA with Tukey post test was applied and P < 0.05 was considered to be statistically significant. Experiments were performed at least three times and data presented are of one representative experiment.

### Immunoblotting and densitometric analysis

Cells (1 × 10^6^) were plated in T25 tissue culture flasks and were treated with ATO or DCA for 12 hours. Cell lysates were prepared by the addition of 500 μl of the M-PER^® ^mammalian protein extraction reagent (Thermo Scientific, IL, USA). Immunoblotting was performed as described previously [25], using the antibodies for c-Myc (Roche, IN, USA, clone 9E10), HIF-1α (Abcam, Cambridge, UK, #ab82832), Bcl-2 (Abcam #ab692), ATP synthase β-subunit (Abcam #ab14730) and β-actin (Abcam #ab8227). Western blots were detected using chemiluminescence and exposure of x-ray film. Images were acquired using a CanoScan 8600F flatbed scanner, and quantitated using the ImageJ software (version 1.4, NIH, USA) and standardized to β-actin in each lane. Results were pooled from 3 separate experiments and expressed as mean ± S.D (n = 3), calculations were performed using the Prism software package, ANOVA with Tukey post test was applied and P < 0.05 was considered to be statistically significant.

## Results

### DCA and ATO together are more effective at reducing cell proliferation and inducing cell death

**Figure 1:**
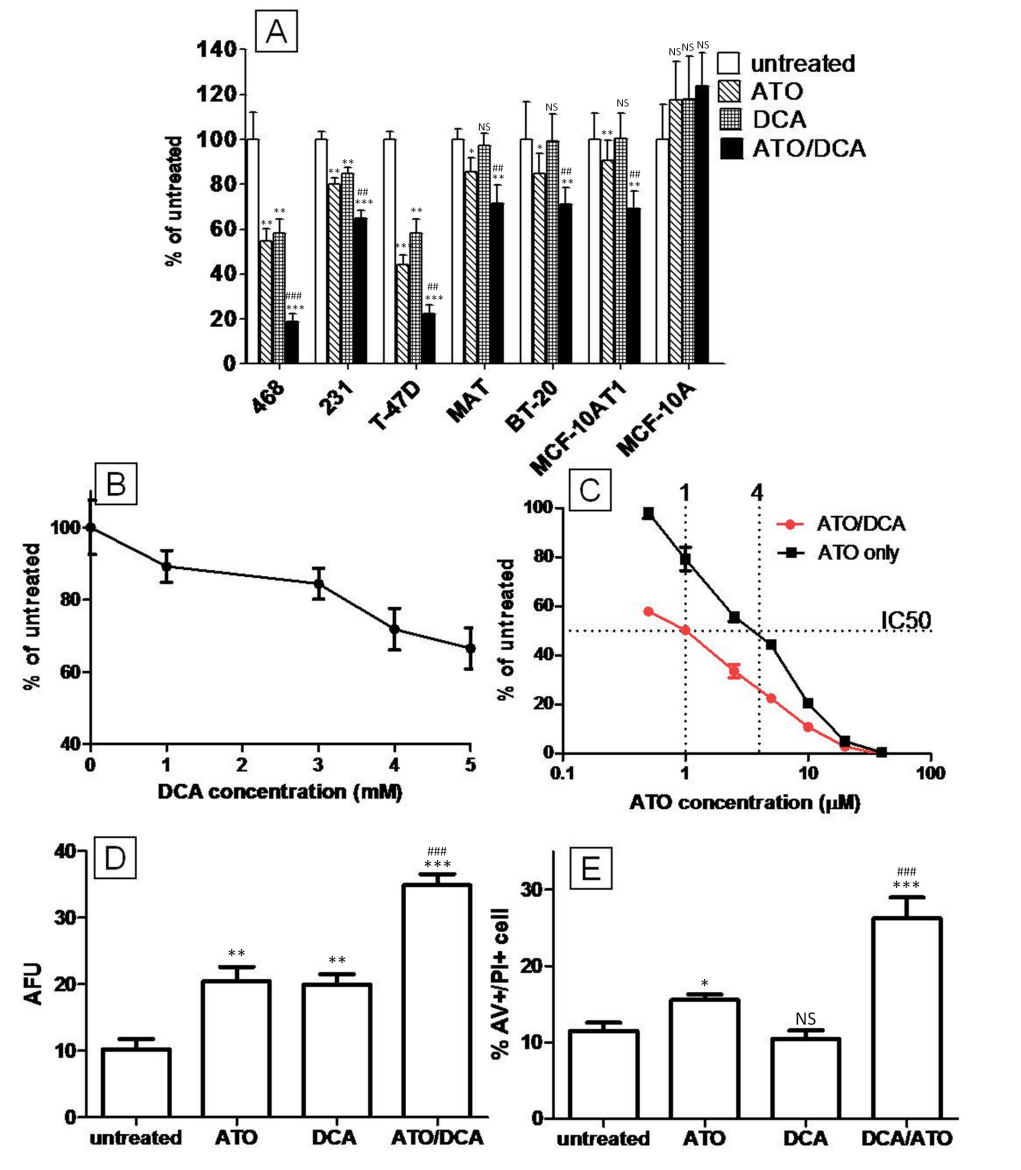
**Inhibition of cancer cell growth by ATO and DCA**. **(A) **Combined effect of ATO (5 or 15 μM) and DCA (5 mM) on breast cancer cell growth as measured by neutral red viability assay after 3 days of treatment. BT-20, T-47D, MDA-MB-468 and MCF-10AT1 cells were treated with 5 μM ATO. MDA-MB-231, MAT and MCF-10A cells were treated with 15 μM ATO. **(B) **Dose response of T-47D cells towards DCA (t = 72 hours). **(C) **Dose response of T-47D cells towards ATO and ATO/DCA (5 mM) treatment (t = 72 hours). **(D) **T-47D proliferation measured as mean CFSE intensity after treatment with 5 μM ATO and/or 5 mM DCA (t = 72 hours) (higher CFSE intensity indicating less proliferation). **(E) **Cell death (AV+/PI+) population expressed as percentage of total cell number after 48 hours of treatment with 20 μM ATO and/or 5 mM DCA. * *P *≤ 0.05, ** *P *≤ 0.001, *** *P *≤ 0.0001, NS - not significant (treated vs untreated). ## *P *≤ 0.001, ### *P *≤ 0.0001 (ATO/DCA vs ATO).

To investigate the combined effect of ATO and DCA at inhibiting cell growth, breast cancer cell lines were treated for several days with both drugs and total cell number was assessed using the neutral red cell viability assay. The panel of cell lines represent the major subtypes of human breast cancer (luminal (T-47D), basal A (MDA-MB-468, BT-20), basal B (MDA-MB-231)), or have other relevance as experimental models (13762MAT - rat mammary adenocarcinoma sensitive to DCA in vivo [22], MCF10AT1 - malignant derivative of immortalized cells MCF-10A). MDA-MB-468, MDA-MB-231, and T-47D cells all showed a significant reduction ranging from 10% to 40% in total cell number after 72 hours treatment with 5 mM DCA (Figure 1A) while 13762 MAT, BT-20 and MCF-10AT1 cells did not respond during the treatment period. All of the cancer cell lines tested were sensitive to ATO but at different concentrations. A reduction in total cell number was seen in BT-20, T-47D, MCF-10AT1 and MDA-MB-468 with only 5 μM ATO treatment, however 15 μM ATO was required to achieve the same efficiency in MDA-MB-231 and 13726 MAT cell lines. Strikingly, DCA and ATO in combination showed a greater effect than either drug alone in all of the cancer cell lines tested. Where ATO and DCA were effective as single agents at reducing cell numbers, the effect of combined treatment was approximately equal to the sum of the individual drug effects (T-47D, MDA-MB-468 and MDA-MB-231). Where DCA alone showed no reduction in cell number, it was still able to enhance the growth inhibition of ATO by 2-3 fold (13762 MAT, BT-20 and MCF10AT1) (Figure 1A). The non-cancerous cell line, MCF-10A showed no reduction in cell number after ATO (15 μM), DCA (5 mM) or combined treatment.

The effect of ATO and DCA was examined further with T-47D cells, one of the cell lines more sensitive to DCA alone. The response of T-47D cells towards DCA was dose-dependent and after 72 hours, 5 mM DCA treated cells had 42 ± 6% less cells than the control culture (Figure 1B). ATO alone (5 μM) reduced the total cell number by 56 ± 4% and cells treated with both DCA and ATO showed a further decrease compared to the ATO only group (Figure 1C). The dose response curve showed that combination treatment of DCA (5 mM) and ATO can reduce the IC_50 _to 0.25-fold that of ATO alone (Figure 1C). This effect is occurring within the concentration range achieved clinically for ATO (up to 5-7 μM [26]).

CFSE proliferation assay demonstrated that either ATO (5 μM) or DCA (5 mM) treated cells emitted significantly higher CFSE fluorescence (2.1-fold and 2.2-fold respectively) after 72 hours of treatment indicating growth inhibition. Cells treated with both ATO and DCA showed a 3.4-fold increase in CFSE intensity compared to untreated cells indicating the drugs worked together at inhibiting cell proliferation (Figure 1D).

The effect of ATO and DCA on T-47D cell death was assessed using AV and PI double staining and cells were analysed using fluorescent cell sorting. DCA alone (5 mM) did not induce cell death in T-47D cells (Figure 1E), similar to the effect of DCA on 13762 MAT cells reported previously [22]. ATO (5 μM) also failed to induce cell death after 12 hours of treatment, however cells treated with both 5 μM ATO and 5 mM DCA showed a small (15%) increase in AV+/PI+ population (13.2 ± 0.6% apoptotic cells compared to 11.5 ± 1.0% for ATO/DCA vs. untreated respectively, p = 0.07), suggesting that DCA may be enhancing the apoptotic effects of ATO. At higher concentrations and with 48 hours of treatment, ATO (20 μM) increased the amount of cell death by 35 ± 8% (*P *= 0.029) compared to the untreated culture (Figure 1E). Combining 5 mM DCA with 20 μM ATO treatment resulted in a 4-fold greater increase in the AV+/PI+ population compared to ATO alone, indicating that DCA can potentiate the cell death induced by ATO in T-47D breast cancer cells (Figure 1E).

### ATO and DCA work together on depolarising the MMP but have counter effects on inducing ATP and ROS production

**Figure 2:**
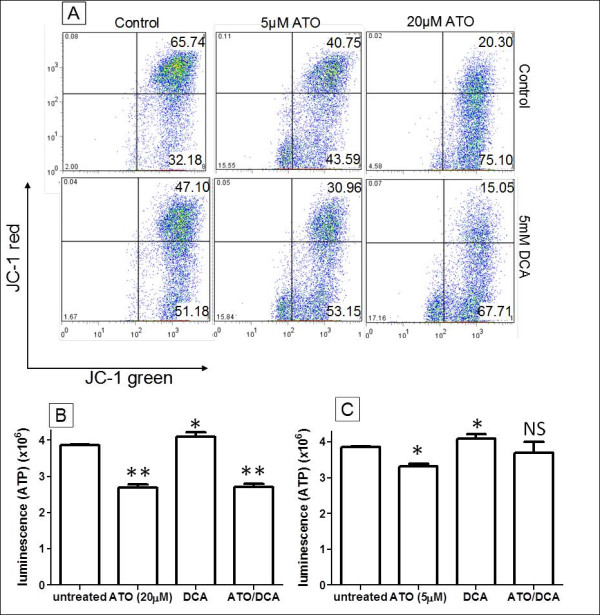
**Intracellular responses of T-47D cells to ATO and DCA treatment**. **(A) **JC-1 staining showing the combined effect of DCA and ATO at depolarizing the MMP (t = 12 hours). **(B and C) **The effects of ATO (20 μM **(B) **or 5 μM **(C)) **and DCA (5 mM) on ATP levels. * *P *≤ 0.05, ** *P *≤ 0.001, NS - not significant (treated vs untreated).

Both ATO and DCA have been shown to alter mitochondrial function, depolarizing MMP and increasing ROS production [20]. Therefore these parameters along with ATP level were studied in an effort to determine whether they contribute to the enhanced anti-cancer effects of ATO/DCA combined treatment. Measured by JC-1 staining, the number of T-47D cells with a hyperpolarized MMP (upper right quadrant) was significantly decreased by DCA (5 mM) or ATO (5 μM and 20 μM) treatment (t = 12 hours) (28 ± 6%, 38 ± 4% and 69 ± 7% decrease respectively compared to untreated control cells), consistent with previously published data [20,27]. Combining ATO (5 μM or 20 μM) with 5 mM DCA lead to an even greater decrease (53 ± 9% and 77 ± 12% respectively) compared to the untreated cells (Figure 2A), demonstrating that DCA and ATO can work together in depolarizing the MMP. ATP levels were decreased by both low and high dose ATO (14 ± 3% and 32 ± 5% respectively) after 12 hours of treatment, while DCA treated cells showed a 6 ± 1% increase in ATP levels (Figure 2B and 2C). At the high dose of ATO, DCA was unable to increase ATP production (Figure 2B), whereas at the low dose of ATO, DCA and ATO combined treated cells showed a small increased in ATP production over ATO treatment alone (Figure 2C). This data indicates that DCA and ATO are affecting ATP production via separate targets inside the cells.

**Figure 3:**
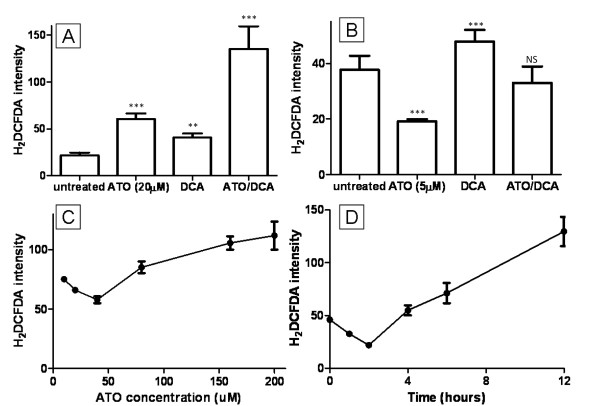
**Effects of ATO and DCA on ROS production**. **(A and B) **The effects of ATO and DCA on ROS levels in T-47D cells after either 20 μM (**A**) or 5 μM (**B**) ATO treatment, alone and in combination with 5 mM DCA. **(C) **Dose response of ROS levels towards different concentrations of ATO (t = 12 hours). **(D) **Time course of ROS production after 10 μM ATO treatment. ** *P *≤ 0.001, *** *P *≤ 0.0001, NS - not significant (treated vs untreated).

Increased intracellular ROS levels have been proposed to be the reason behind ATO cytotoxicity [2,9]. The combined effect of ATO and DCA on ROS was investigated using the fluorescent stain H_2_DCFDA. Cells treated with 20 μM ATO or 5 mM DCA showed elevated intracellular ROS levels (2.8 and 1.9-fold respectively) (Figure 3A) similar to previously published results [27], while cells treated with both ATO and DCA showed a further increase (5.2-fold) in ROS production.(Figure 3A). In contrast, low concentration ATO (5 μM) induced a 0.5-fold decrease in ROS production (Figure 3B). This was contrary to previous reports on ROS production and ATO treatment, so the dose-response and time course of ROS production after ATO treatment was analysed. This revealed that lower concentrations of ATO decreased intracellular ROS production while high ATO induced ROS production (Figure 3C). Similarly 10 μM ATO treatment resulted in a notable decrease in ROS production in the first 4 hours before ROS levels increased after 8 hours to the level reported by others (Figure 3D). Combined treatment of cells with 5 mM DCA and 5 μM ATO resulted in an intermediate ROS production (Figure 3B) similar to untreated cells.

**Figure 4:**
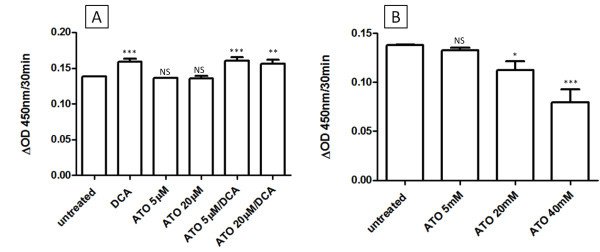
**Effects of ATO and DCA on PDH activity**. **(A) **The effects of on PDH activity in T-47D cells after 3 hr of treatment with ATO and DCA. **(B) **Effect of ATO treatment on the PDH activity of isolated PDH (antibody capture from T-47D cells). * *P *≤ 0.05, ** *P *≤ 0.001, *** *P *≤ 0.0001, NS - not significant (treated vs untreated).

The intermediate effects of DCA/ATO combination treatment on ATP and ROS levels may be explained by competing effects on PDH activity. DCA targets PDK, and therefore should increase PDH activity, whereas ATO has been reported to inhibit PDH either directly by reaction with vicinal thiols on PDH or indirectly via increased hydrogen peroxide production [28]. To examine the effects of ATO and/or DCA on PDH activity, intact cells or PDH isolated from T-47D cells, were treated with drug and the PDH activity determined. As predicted, 3 hr treatment of intact cells with DCA resulted in increased PDH activity, whereas ATO (up to 20 μM) did not alter PDH activity of intact cells (Figure 4a). In contrast, DCA did not change the activity of isolated PDH, but high concentrations of ATO were able to inhibit PDH activity (Figure 4b). Thus in T-47D cells, ATO did not decrease PDH activity at the concentrations used in this study.

### ATO is an inhibitor of complex IV of the electron transport chain

**Figure 5:**
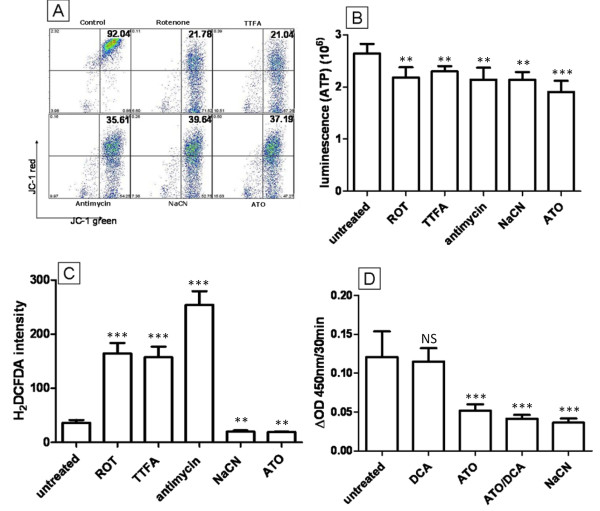
**Inhibition of the ETC by ATO**. **(A to C) **The effects of a series of ETC inhibitor treatments were compared to 5 μM ATO (t = 12 hours): **(A) **MMP by JC-1 staining; **(B) **ATP levels; **(C) **ROS levels. **(D) **Cytochrome C oxidase activity after treatment (5 min) with DCA (5 mM) and/or ATO (5 μM). ** *P *≤ 0.001, *** *P *≤ 0.0001, NS - not significant (treated vs untreated).

The electron transport chain (ETC) is located in the inner membrane of the mitochondria and plays a vital role in energy production. The ETC is responsible for generating the proton gradient for the inner membrane space of mitochondria to maintain the MMP for ATP production [29]. The ETC is also documented as the major site of ROS production within cells as a result of electron leakage from complex I and III [30]. Due to its ability to lower ROS, depolarize MMP and decrease ATP production simultaneously, we have hypothesised that ATO operates as an ETC inhibitor. A panel of ETC inhibitors was compared to ATO (5 μM) to identify whether they induce the same phenotypic changes as ATO in T-47D cells. Rotenone (0.1 μM, complex I), thenolytrifluoroacetone (TTFA) (10 μM, complex II), antimycin (0.1 μM, complex III) and NaCN (10 mM, complex IV) were used to treat T-47D cells and ROS, MMP and ATP levels were compared to ATO treated cells. All of the inhibitors demonstrated abilities to depolarize MMP (Figure 5A) and reduce ATP levels (Figure 5B) similarly to ATO. However, in contrast to ATO, rotenone, antimycin and TTFA failed to reduce ROS production within cells, instead elevating ROS 3- to 6-fold after 12 hours treatment (Figure 5C). Cells treated with NaCN (10 mM) however, showed a 52% decrease in ROS production (Figure 5C) similar to ATO treatment. Based on these data we concluded that in T-47D breast cancer cells, the inhibition of complex IV of the ETC but not complexes I-III results in a reduction of ROS, and therefore it is likely that phenotypic changes induced by ATO are caused by the inhibition of complex IV (cytochrome C oxidase) of the ETC. To confirm this, cytochrome C oxidase activity in T-47D cells was measured spectrophotometrically. Cytochrome C oxidase assay clearly demonstrated that while DCA had no effect on complex IV activity, ATO (5 μM, 5 min) can inhibit complex IV activity confirming our hypothesis (Figure 5D). Similar results were obtained after 10 min, 3 hr and 12 hr drug treatments. The combination of ATO and DCA showed similar enzyme inhibition to ATO alone.

### Mitochondrial transition pore inhibitors partially blocked ATO induced caspase activation but had no effect on ROS, ATP and MMP

**Figure 6:**
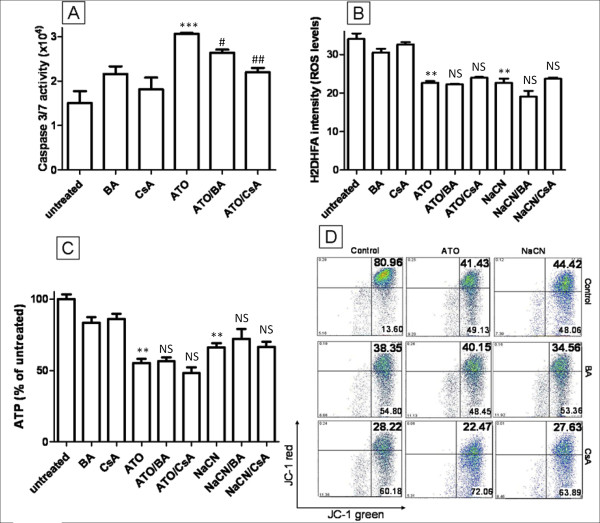
**Effect of the MTP blockers CsA and BA on ATO treated cells**. **(A) **CsA and BA can both partially block 20 μM ATO induced caspase 3/7 activation. **(B to D) **In contrast, CsA and BA both failed to block the NaCN or 5 μM ATO induced reduction in ROS (**B**), reduction in ATP levels (**C**) and depolarization of the MMP (**D**). * *P *≤ 0.05, ** *P *≤ 0.001, *** *P *≤ 0.0001 (treated vs untreated). # *P *≤ 0.05, ## *P *≤ 0.001 (compared to ATO alone). NS - not significant compared to ATO or NaCN alone.

One mechanism that has previously been described as the key to ATO cytotoxicity is the opening of the mitochondrial transition pore (MTP). ATO has been shown to induce the opening of the MTP in isolated mitochondria and can increase cytochrome C release [11] and induce nuclear apoptosis in cell free systems [31]. The opening of MTP by ATO has been proposed to induce mitochondrial permeabilization, dissipate the MMP and increase intracellular ROS. To test whether the MTP is important for ATO toxicity in T-47D cells, bongkrekic acid (BA) and cyclosporine A (CsA), both potent MTP pore blockers [32], were combined with ATO to see whether they can inhibit ATO induced intracellular changes. The addition of 5 μM CsA or 50 μM BA to ATO (5 μM) treated cells did not interfere with the ability of ATO to lower ROS production after 12 hours of treatment, decrease ATP production or depolarise the MMP (*P *> 0.05) (Figure 6B, C and 6D). Similar data can be seen for NaCN and CsA/BA treatment (*P *> 0.05) (Figure 6B, C and 6D). To confirm the activity of CsA and BA in our experimental conditions, caspase 3/7 activity, an apoptosis marker downstream of MTP opening, activated by cytochrome C release, was assessed after high concentration ATO treatment (Figure 6A). ATO treatment (20 μM) doubled caspase 3/7 activity compared to untreated cells (2.1-fold, Figure 6A) and this effect was decreased by BA and CsA (1.7-fold and 1.4-fold, respectively), indicating that CsA or BA can block MTP opening in T-47D cells. These data clearly demonstrate that although ATO (20 μM) can induce the opening of MTP, this mechanism cannot explain the ROS, MMP and ATP changes occurring during 5 μM ATO treatment. We conclude that these alterations are likely due to the inhibition of ETC complex IV.

### DCA and ATO have opposing effects on ATP synthase β-subunit and Bcl-2 expression while working co-operatively at down-regulating c-Myc and HIF-1α protein levels

**Figure 7:**
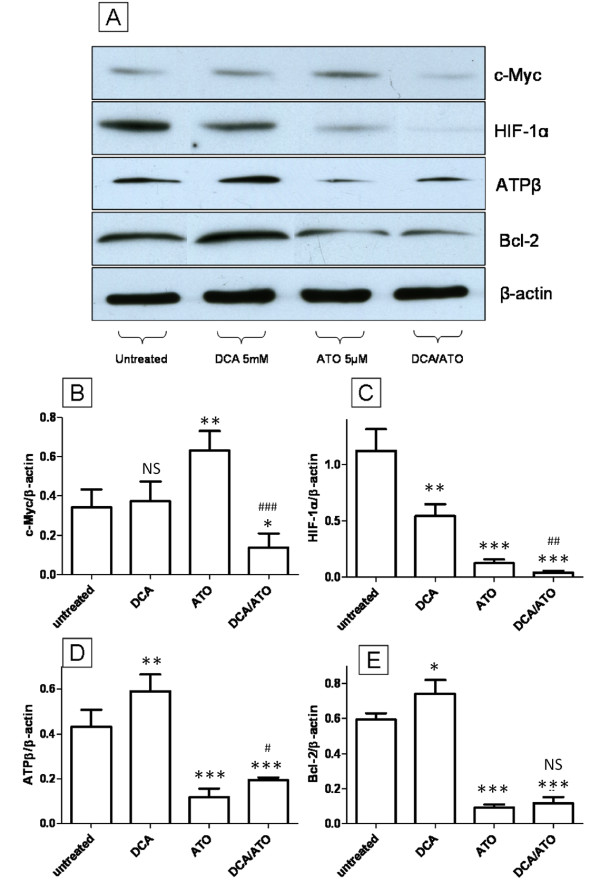
**Protein expression changes after ATO and DCA treatment**. **(A) **Immunoblotting of T-47D cells treated with ATO and/or DCA. **(B-E) **Densiometric analysis for c-Myc **(B)**, HIF-1α **(C)**, ATPβ **(D)**, Bcl-2 **(E) **and β-actin (control) was done on blotting of whole cell lysates prepared from T-47D cells treated with 5 mM DCA and/or 5 μM ATO for 12 hours. * *P *≤ 0.05, ** *P *≤ 0.001, *** *P *≤ 0.0001, NS - not significant (treated vs untreated). # *P *≤ 0.05, ## *P *≤ 0.001, ### *P *≤ 0.0001 (DCA/ATO vs ATO).

We examined the effect of 5 mM DCA and 5 μM ATO treatment (12 hours) on the expression c-Myc and HIF-1α, two major transcription factors that are known to regulate the Warburg effect [33] and mitochondrial activity [34], and on the expression of the mitochondrial proteins ATP synthase β subunit (ATPβ) and Bcl-2. Immunoblotting for c-Myc showed no change after treatment with DCA while ATO induced the up-regulation of c-Myc (Figure 7A and 7B), however ATO in combination with DCA reduced c-Myc level compared to the untreated control. HIF-1α levels on the other hand showed a down regulation after both DCA and ATO treatment. The combination of ATO and DCA showed further down-regulation of HIF-1α compared to single agent treated and control groups (Figure 7A and 7C). Bcl-2 is a member of the BH3 protein family that binds directly to Bad and Bax and prevents apoptosis [35]. Immunoblotting performed for ATPβ found that DCA treatment up-regulated ATPβ while ATO showed an opposite effect, lowering ATPβ levels (Figure 7A and 7D), and ATO and DCA combined treatment showed an intermediate response compared to ATO or DCA treated groups. Immunoblotting for the pro-survival protein Bcl-2 showed an up-regulation after DCA, down-regulation for ATO treatment, and an intermediate response from the combination of the two drugs (Figure 7A and 7E). These results are also very similar to ATP levels and ROS production where DCA and ATO had opposing effects (Figure 2C and 3B). The Bcl-2 and ATPβ results directly correlate with DCA and ATO's ability to influence ROS production and ATP levels.

## Discussion

During the last decade, ATO has been used effectively to treat both newly diagnosed and relapsed APL patients, with patients showing complete remission after low dose ATO treatment [4]. The anti-cancer ability of ATO is not limited to APL [36] and many other tumors in animal models have been shown to be sensitive to ATO treatment [37,38]. However, the lack of information on sites of action for ATO cytotoxicity in tumor types other than APL has limited its uptake for the treatment of other cancers until recent years [5].

In this study, we have demonstrated that complex IV of the ETC is a target for ATO. From a panel of ETC inhibitors, the decrease in ROS, decrease in ATP production and MMP depolarisation caused by low dose ATO treatment of T-47D cells was reproduced only by cyanide, a well characterised complex IV inhibitor (Figure 5). Direct measurement of cytochrome C oxidase activity in whole cells treated with ATO confirmed the ability of ATO to inhibit this enzyme activity directly. The cytochrome C oxidase complex of the ETC is known to have closely spaced cysteine residues [39], and these are likely to react with ATO.

Most cancer cells have up regulated ROS levels compared to normal cells [40] and the ETC is considered the major source of intracellular ROS. The reduction in ROS of up to 60% by NaCN or ATO, demonstrated that complex IV is responsible for the majority of ROS production in T-47D cells. The oxidative stress of elevated ROS production has been described as a double edged sword. A moderate increase in ROS has been shown to be associated with increased proliferative potential [41], increased antioxidant enzymes such as glutathione transferase [42], superoxide dismutase and catalase [43] and increased pro-survival proteins such as Bcl-2 [42] and survivin [44]. However, intolerable amounts of ROS eventually leads to cell death [45]. The reduction in ROS after 12 hours treatment of 5 μM ATO also directly correlated with a decrease in Bcl-2 levels (Figure 7E) suggesting that T-47D cells are in an apoptotic resistant state due to elevated ROS levels. Continued inhibition of complex IV of the ETC will lead to the depolarisation of the MMP and inhibition of ATP production. The cell death that follows from this mitochondrial dysregulation will give rise to the elevated ROS seen after 4 hours or at higher ATO concentration (Figure 3C and 3D) as reported by others [46] after ATO treatment. Thus elevated ROS is a consequence, not the cause, of ATO induced cell death.

The previously proposed mitochondrial target for ATO is the MTP. Zheng et al demonstrated that ATO induces the opening of the MTP in isolated rat liver mitochondria [11], leading to the proposal that MTP is responsible for the release of ROS and the depolarization of the MMP seen during arsenic treatment, and subsequent apoptosis [47]. Our study does not exclude MTP as a target for ATO. Our data indicate that inhibition of the MTP via CsA and BA can partially block caspase 3/7 activation induced by high concentration ATO, however the MTP blockers failed to inhibit the depolarization of MMP and the reduction in ROS and ATP levels caused by low concentration ATO. Thus although ATO can open the MTP, alternative targets are required to explain the action of ATO at low concentrations. Inhibition of the thioredoxin system has been shown to occur in MCF-7 cells after treatment with 2-5 μM ATO and may mediate some cellular effects of ATO at low concentrations [13], however this mechanism would lead to increased oxidative stress, and cannot explain the decreased ROS observed in the current study.

DCA, as a pyruvate dehydrogenase kinase inhibitor, can reverse the glycolytic effect and has demonstrated significant anti-cancer properties both *in vitro *and *in vivo *[17-23]. Some studies show increased apoptosis with DCA [17,18,20]. However our studies in breast cancer and studies by Stockwin et al indicate that DCA acts as a cytostatic rather than cytotoxic agent across a range of cell lines except at very high concentrations [22,48]. Michelakis recently reported *trough *serum levels of DCA to be 0.5 mM in glioblastoma patients taking 6.25 mg/kg orally twice daily [23], thus the clinically relevant concentrations are likely to be in the range of 0.5-5 mM. DCA has been shown to induce a small but significant increase in caspase 3/7 activity even when apoptosis was not observed and it was proposed that DCA may sensitize cancer cells towards cytotoxic agents [22]. The current study confirmed this hypothesis by showing that DCA sensitized T-47D cells towards ATO treatment as seen from AV+/PI+ double staining (Figure 1E and text). ATO was selected as the agent for testing due to its anti-mitochondrial properties, as we proposed that by reversing the glycolytic phenotype with DCA, and directing more pyruvate into mitochondrial oxidative phosphorylation, while simultaneously targeting the mitochondria with ATO, a cooperative effect would be seen with this drug combination. The principle of this dual targeting strategy is supported by the results of two recent publications which found that cells with mitochondrial defects (e.g. rho (0) or after treatment with non-drug mitochondrial inhibitors such as rotenone) show higher sensitivity to the growth inhibition effects of DCA [48,49]. While the efficacy of this dual targeting strategy is yet to be demonstrated in vivo, the ATO/DCA enhanced combination effects were seen on both growth inhibition and apoptosis at drug concentrations in clinically relevant ranges. Some effects at the lower end of the concentration range were not large (eg. 15% increase in apoptotic cells), however the impact *in vivo *over weeks rather than hours of treatment warrants further investigation.

It has been well documented that DCA can alter mitochondrial behavior i.e. depolarize the MMP [18,20]. However this phenomenon has not been well explained. We observed that DCA can increase ATP while depolarizing MMP in T-47D cells (Figure 2). Based on these data we proposed that DCA is altering mitochondrial function through complex V of the ETC, ATP synthase. ATP synthase is the last step of the ETC and it uses the MMP generated by the ETC to produce ATP. ATPβ is down-regulated in lung, brain, breast and gastric cancers [50], which could explain the hyperpolarized mitochondrial membrane that can be found in many cancer cells [51]. We have hypothesized that DCA can increase ATP synthase activity either via allosteric regulation due to its homology to pyruvate or via increase in ATP synthase protein levels after the reversal of the Warburg effect and decreased HIF-1α levels. Immunoblotting clearly demonstrated that ATPβ is up-regulated after DCA treatment (Figure 7D) suggesting that DCA can increase ATP synthase activity, which in turn contributes to increased ATP production and exhaustion of the MMP.

HIF-1α and c-Myc are two major oncogenic transcription factors known to regulate metabolism in cancer cells. HIF-1α can up regulate the enzymes of the glycolytic pathway and lactic acid dehydrogenase, sustaining the Warburg effect in cancer cells [33]. This not only increases ATP production, but also increases the supply of precursors such as glucose-6-phophate and fructose-6-phosphate for the production nucleic acids through the pentose phosphate pathway [16]. c-Myc, on the other hand, not only plays a central role in promoting G1 to S phase cell cycle transition by regulating the cyclins and their kinases and inhibitors, but also up regulates protein components of the ETC such as COXI-IV and helps to increase mitochondrial activity [52]. Thus, the combination of c-Myc and HIF-1α overexpression is important for inducing the Warburg effect while increasing mitochondrial activity, supporting the glutamine-TCA hub that is essential for anabolism of amino acids and fatty acids needed for cell division [16]. This metabolic signature contributes to carcinogenesis and the malignant phenotype of many tumors [33,34]. Both ATO and DCA can decrease HIF-1α levels significantly suggesting a reduction in the Warburg effect (Figure 7C). While DCA alone had no effect on c-Myc levels, surprisingly, ATO up regulated c-Myc levels significantly (Figure 7B). This may be a positive feed back loop, where the cells are trying to increase ETC activity after the inhibition of complex IV. Strikingly, DCA reversed the effect of ATO on c-Myc, and the combination of ATO/DCA strongly repressed c-Myc expression, correlating with the ability of these agents to work together at reducing cell proliferation and inducing cell death (Figure 1).

The efficacy of ATO against PML has been attributed to several special features of this malignancy - differentiation through inactivation of PML-RARα, low antioxidant capacity of PML cells to protect against the elevated ROS levels, and accumulation of the drug due to impaired osmoregulation [5]. Many trials are underway for the use of ATO in a range of solid malignancies, although thus far there are few results published. Results that are available suggest that ATO is not highly effective as a single agent in patients with advanced cancer of the pancreas, liver or melanoma [5]. Investigators are advocating evaluating ATO in combination with other anticancer drugs [53,54] with some phase I trials being completed [55]. As our understanding of the mechanisms of ATO continues to grow, especially its effects through cancer metabolism, we may be better able to harness its anticancer potential *in vivo *in novel drug combinations.

## Conclusions

Two recent reports found DCA to be most effective against cells with mitochondrial defects [48,49]. This report is the first to demonstrate that targeting of two aspects of metabolism - reversing of the Warburg effect with DCA while inhibiting oxidative phosphorylation with ATO - is clearly an effective anti-cancer strategy in vitro against breast cancer cell lines. The identification of cytochrome C oxidase as a mitochondrial target for ATO provides novel mechanistic information for the application of ATO to the treatment of tumor types other than APL. The ability of DCA to increase ATP synthase β subunit expression, reversing another widespread cancer metabolic phenotype, also suggests DCA may be relevant to a broad range of tumor types. The ability of DCA to enhance the cytotoxic effects of chemotherapeutic agents other than ATO *in vitro *and *in vivo *is also worthy of further investigation. The lack of sensitivity of the non-cancerous cell line MCF-10A to the clinically relevant concentrations of ATO/DCA tested is also encouraging, and suggests that this treatment strategy should be further tested against solid tumors *in vivo*.

## List of abbreviations

APL: acute promyeloid leukemia; ATO: arsenic trioxide; ATPβ: ATP synthase β subunit; AV: annexin V; BA: bongkrekic acid; CFSE: carboxyfluorescein succinimidyl ester; CsA: cyclosporine A; DCA: dichloroacetate; ETC: electron transport chain; H_2_DCFDA: 2', 7'-dihydrochlorofluroresceinacetate; JC-1: 5,5',6,6'-tetrachloro-1,1', 3,3'-tetraethylbenzimidazol-carbocyanine iodide; MMP: mitochondrial membrane potential; MTP: mitochondrial transition pore; NaCN: sodium cyanide; PI: propidium iodide; ROS: Reactive oxygen species; TTFA: thenolytrifluoroacetone.

## Competing interests

The authors declare that they have no competing interests.

## Authors' contributions

RS designed and carried out the experiments, and drafted the manuscript. PB participated in the analysis of data and manuscript preparation. AB participated in the design and analysis of the data, and prepared the manuscript for publication. All authors have read and approved the final manuscript.
